# Short-Term Exposure to Thermophilic Temperatures Facilitates CO Uptake by Thermophiles Maintained under Predominantly Mesophilic Conditions

**DOI:** 10.3390/microorganisms10030656

**Published:** 2022-03-18

**Authors:** Caitlin K. Wilson, Gary M. King

**Affiliations:** Department of Biological Sciences, Louisiana State University, Baton Rouge, LA 70803, USA; kingcaitline@gmail.com

**Keywords:** carbon monoxide oxidation, thermophile, mesophile, variable temperature

## Abstract

Three phylogenetically and phenotypically distinct CO-oxidizing thermophiles (*Alicyclobacillus* *macrosporangiidus* CPP55 (Firmicutes), *Meiothermus* *ruber* PS4 (Deinococcus-Thermus) and *Thermogemmatispora* *carboxidovorans* PM5^T^ (Chloroflexi)) and one CO-oxidizing mesophile (*Paraburkholderia paradisi* WA^T^ (Betaproteobacteria)) isolated from volcanic soils were used to assess growth responses and CO uptake rates during incubations with constant temperatures (25 °C and 55 °C) and during multi-day incubations with a temperature regime that cycled between 20 °C and 55 °C on a diurnal basis (alternating mesophilic and thermophilic temperatures, AMTT). The results were used to test a conjecture that some thermophiles can survive in mesothermal habitats that experience occasional thermophilic temperatures. *Meiothermus* *ruber* PS4, which does not form spores, was able to grow and oxidize CO under all conditions, while the spore-forming *Alicyclobacillus* *macrosporangiidus* CPP55 grew and oxidized CO during the AMTT regime and at 55 °C, but was not active at 25 °C. *Thermogemmatispora* *carboxidovorans* PM5^T^, also a spore former, only grew at 55 °C but oxidized CO during AMTT and 55 °C incubations. In contrast, the non-sporing mesophile, *Paraburkholderia paradisi* WA^T^, was only able to grow and oxidize CO at 25 °C; growth and CO uptake ceased during the AMTT incubations after exposure to the initial round of thermophilic temperatures. Collectively, these results suggest that temporary, periodic exposure to permissive growth temperatures could help maintain populations of thermophiles in mesothermal habitats after deposition from the atmosphere or other sources.

## 1. Introduction

Thermophilic bacteria possess multiple adaptations that enable growth at temperatures above 50 °C [1]. These adaptations include, among others, modified membrane lipid contents, altered %G+C contents, and modifications of amino acid compositions that promote enzyme stability and activity [2,3,4,5]. Although they are adapted to temperatures found most commonly in hot springs and other geothermally heated systems, numerous studies of a wide variety of mesothermal, and even cold, habitats have shown that thermophiles are not confined to sites with elevated temperatures [6,7,8,9,10,11,12,13,14,15,16,17]. In some instances, thermophiles have even been isolated from sites that are permanently cold with temperatures well below permissive ranges reported for thermophilic growth.

The ubiquitous distribution of thermophiles in a predominantly mesothermal world has long been considered enigmatic. Several non-exclusive possibilities have been offered to account for their distribution. Hubert et al. [18] and Perfumo and Marchant [19] have proposed that continuous inputs of thermophiles from various sources (e.g., the atmosphere) offset losses due to mortality and maintain essentially dormant populations in a quasi-steady state. In essence, recruitment equals loss.

In contrast, Portillo et al. [20] and Cockell et al. [21] have suggested that thermophiles in mesothermal habitats might occasionally experience elevated temperatures in situ that permit limited metabolic activity, or even growth. For example, Portillo et al. [20] found that thermophilic Firmicutes made up 3.4% of the active microbial community of a Spanish soil. This soil experienced a broad diurnal temperature regime that included values permissive for some thermophiles (25–60 °C; >45 °C, 7 h d^−1^). Temporary exposures to elevated temperatures at this site might have been enough to sustain stable and metabolically active thermophilic populations. Similarly, Cockell et al. [21] observed daily maximum temperatures up to 44.5 °C for a basalt formation in Iceland, where the average annual temperature was only 10 °C; they suggested that this could be sufficient to support limited activity by thermophiles.

In addition to numerous isolation-based studies, thermophilic microbial activity has been documented for a variety of mesothermal soils and sediments. In particular, carbon monoxide (CO) uptake has been observed under oxic and anoxic conditions for soils and sediments incubated at thermophilic temperatures (e.g., [16,17,22]). Aerobic thermophilic CO oxidizers have also been isolated from temperate sites that experience small (15–25 °C) and large (15–55 °C) diurnal temperature fluctuations [23,24]. These isolates include spore formers and non-spore-formers, representing the Firmicutes, Chloroflexi (Ktedonobacteria) and Deinococcus–Thermus phyla.

Results reported here provide a first cultivation-based test of the concept that exposure to conditions that include mesophilic and thermophilic temperature regimes can maintain active populations of thermophiles even when temperature regimes are largely non-permissive. To that end, growth and CO oxidation by three phylogenetically and phenotypically distinct CO-oxidizing thermophiles were monitored during incubations at 25 °C, 55 °C or during a cycle that varied diurnally between 20 °C and 55 °C to mimic ambient temperature regimes observed in situ at a field site [22]. Each of the three thermophilic isolates oxidized CO and two of the three grew during the variable temperature cycle. These outcomes established the potential for at least some thermophiles to remain metabolically active in mesothermal habitats that intermittently experience temperatures up to 55 °C. The results also suggest that some soil thermophiles might play as yet unappreciated roles in soil–atmosphere trace gas exchanges during periods with elevated temperatures and that these roles might increase in a warm world with more frequent extreme heating events.

## 2. Materials and Methods

### 2.1. CO-Oxidizing Isolates

Three thermophilic CO oxidizers (two spore forming and one non-sporing) and one non-sporing mesophilic CO-oxidizing isolate were assessed for their responses to incubation temperatures that included constant mesophilic or thermophilic regimes, as well as alternating mesophilic–thermophilic regimes (AMTT). All isolates were obtained during prior studies of mesophilic and thermophilic CO-oxidizing bacteria isolated from sites on Kilauea Volcano, Hawaii (USA). Methods for isolation, characterization and analyses of 16S rRNA genes and form I CO dehydrogenase (*coxL*) sequences have been reported previously (e.g., [22,23,25]).

Briefly, *Alicyclobacillus macrosporangiidus* strain CPP55 (DSM 26552), a spore-forming Firmicute, was isolated from soil collected from a forested stand at the Pu‘u Puai tephra field (Hawaii) and incubated in the laboratory at 55 °C. *Alicyclobacillus macrosporangiidus* CPP55 oxidized CO and harbored a *coxL* gene (accession KF193565.1); it was most closely related to *A. macrosporangiidus* strain 5-A239-2O-A^T^ (98.7% 16S rRNA gene sequence identity; accession KF193532.1) but not characterized further. *A. macrosporangiidus* has been reported to grow between 35–65 °C with an optimum from 50–55 °C (Goto et al., 2007).

*Thermogemmatispora carboxidovorans* PM5^T^ (DSM 45816), a spore-forming thermophile, was isolated from microbial mats at the Puhimau Thermal Area (Hawaii); mat temperatures averaged 42 °C with substantial fluctuations, including temperatures up to 65 °C. *T. carboxidovorans* PM5 was described as a novel CO-oxidizing member of the Ktedonobacteria class, with a growth range from 40–65 °C and a growth optimum of 55 °C [22].

*Meiothermus ruber* PS4, a non-sporing member of Deinococcus-Thermus, was obtained from a geothermally heated soil (collection temperature 78 °C) at the Puhimau Thermal Area. This isolate yielded a 16S rRNA gene sequence (accession KF193551.1) that was 100% identical to that of *M. ruber* strain 21^T^; it also harbored a form I *coxL* gene (accession KF193564.1) and oxidized CO aerobically. It was not characterized further, but the type strain for *M. ruber* has been reported to grow between 37–70 °C, with an optimum at 60 °C [26].

*Paraburkholderia paradisi* WA^T^ (DSM 28027), a non-sporing mesophile, was isolated from a Pu‘u Puai forested stand and described as a novel CO-oxidizing member of the Betaproteobacteria by Weber and King [27]. Soils at the Pu‘u Puai site experienced small diurnal temperature fluctuations (15 °C–25 °C). *P. paradisi* grew between 15–45 °C with an optimum at 30 °C.

For this study, *A. macrosporangiidus* CPP55 was cultivated in DSM Medium 13 modified with 10 mg L^−1^MnSO_4_; pH was adjusted to 4.2, which favors *Alicyclobacillus* growth [28]. *M. ruber* PS4, *T. carboxidovorans* PM5 and *P. paradisi* WA were grown in DSM Medium 592 at pH 6.5. Prior to each experiment, isolates were grown in 100 mL of medium in sealed flasks (500 mL) containing air headspaces that were amended with CO (50 ppm); flasks were incubated at 25 °C or 55 °C for mesophilic and thermophilic isolates, respectively. After the cultures reached stationary phase, they were used for the CO uptake and growth assays described below.

### 2.2. Temperature Responses

**Figure 1:**
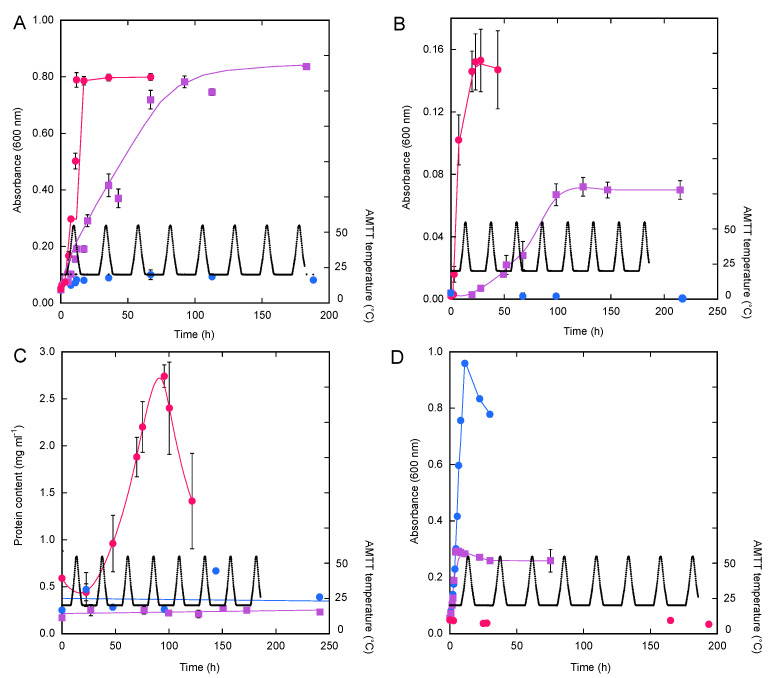
Growth (either A_600_ or protein concentration) of *Meiothermus ruber* PS4 (**A**), *Alicyclobacillus macrosporangiidus* CPP55 (**B**), *Thermogemmatispora carboxidovorans* PM5 (**C**) and *Paraburkholderia paradisi* WA (**D**) at 55 °C (•), with the AMTT regime (■) or at 25 °C (•). AMTT temperatures are indicated in black. Data are means of triplicate determinations with ±1 standard error; some error bars are smaller than the symbols.

Growth responses of the isolates to various temperature regimes (constant mesophilic (25 °C), constant thermophilic (55 °C), and alternating mesophilic–thermophilic temperatures (AMTT)) were assessed after 0.5 mL of a pre-grown culture was used to inoculate each of triplicate 75 cm^3^ serum bottles for each incubation temperature. The bottles contained 9.5 mL of a suitable medium (see above) and an air headspace, and they were sealed with blue butyl rubber stoppers. Triplicates for each isolate and triplicate uninoculated medium controls were incubated with shaking at either 25 °C, 55 °C or with the AMTT program (see Figure 1). The AMTT program was initiated at 20 °C using an Echotherm Model IN45 programmable chilling incubator (Torrey Pines Scientific, Carlsbad, CA, USA) and maintained for 8 or more 24 h cycles, each of which resulted in exposure to a peak temperature of 55 °C once daily (e.g., Figure 1).

Sub-samples (0.75 mL) of *M. ruber* PS4, *A. macrosporangiidus* CPP55 and *P. paradisi* WA replicates were collected using sterile needles and 1 cm^3^ syringes at regular intervals during the incubation for measurements of absorbance (600 nm) using a spectrophotometer (Beckman model DU 640, Beckman-Coulter, Brea, CA, USA). Growth rate constants for the various incubations were determined from the slopes of the log of absorbance plotted versus time. Due to the formation of small aggregates, growth of *T. carboxidovorans* PM5 was determined using cell protein content in lieu of absorbance. Sub-samples (1 mL) of these cultures were obtained at intervals using needles and 1 cm^3^ syringes as before. Cells were harvested by centrifugation (25 °C, 60 s, 15,000× *g*), for which the pellets were lysed in a 5% SDS, 0.1 M NaOH solution with sonication (20 s) then incubated at 95 °C for 15 min. Protein content was determined using a Pierce bicinchoninic acid protein assay kit (Thermo Fisher Scientific, Waltham, MA, USA).

### 2.3. CO Uptake Assays

CO uptake assays were conducted using stationary phase cells in their initial growth medium and washed cells resuspended in a substrate-free minimal medium. To prepare washed cells, a portion (45 mL) of each stationary phase culture was centrifuged twice and resuspended in a minimal medium. To produce a minimal version of medium 13 for *A. macrosporangiidus* CPP55, yeast extract and glucose were omitted. To produce a minimal version of medium 592 for *M. ruber* PS4, *T. carboxidovorans* PM5 and *P. paradisi* WA, yeast extract and tryptone were omitted. Aliquots (5 mL) of unwashed and washed cultures were transferred to 75 cm^3^ serum bottles that were then sealed with gas-tight stoppers; CO (100 ppm) was added to each headspace. Triplicate samples for both unwashed and washed cultures of each isolate and uninoculated media controls were incubated with shaking at 25 °C, with the AMTT program and at 55 °C. CO levels were determined by sampling the culture headspaces with a sterile needle and syringe and analyzing CO concentrations using a gas chromatograph (Trace Analytical Model RGA3, Menlo Park, CA, USA) as previously described [29]. After completing the assays, cells were harvested by centrifugation (25 °C, 15,000× *g*) and cellular protein content was determined as described above.

## 3. Results

### 3.1. Isolate Growth

In this study, isolates were incubated at constant temperatures (25 °C or 55 °C) or with a variable temperature cycle that included mesophilic and thermophilic temperatures (AMTT). Under these conditions, *M. ruber* PS4 grew optimally at 55 °C (0.23 ± 0.01 h^−1^), but it also grew during the AMTT regime (Table 1, Figure 1A), albeit at a twofold lower rate than at 55 °C (0.11 ± 0.01 h^−1^). Growth during the AMTT cycles was essentially continuous, irrespective of the changing temperatures (Figure 1A). Maximum cell densities at the stationary phases for 55 °C and the AMTT incubations were comparable (Figure 1A). Slow, but distinct, growth was also observed at 25 °C (0.05 ± 0.01 h^−1^) but maximum cell densities were much lower than for the other conditions and the stationary phase was reached more quickly than for the AMTT regime (Figure 1A).

*A. macrosporangiidus* CPP55 also grew optimally at 55 °C (0.70 ± 0.10 h^−1^; Table 1) and it grew continuously through multiple AMTT cycles (Figure 1B), though at a slower rate (0.36 ± 0.01 h^−1^) and to a lower maximum cell density than at 55 °C. No growth was observed at 25 °C (Table 1, Figure 1B). In contrast, *T. carboxidovorans* PM5 only grew at 55 °C (Table 1, Figure 1C), with no growth at 25 °C or under the AMTT conditions in spite of an extended incubation period. The mesophile, *P. paradisi* WA, grew optimally at 25 °C (0.33 ± 0.01 h^−1^), but it also grew relatively rapidly during the initial phase of the AMTT regime, though to a lower maximum density (Table 1, Figure 1D). However, no growth was observed during or after the first thermophilic phase of the AMTT regime or during incubation at 55 °C.

### 3.2. Isolate CO Uptake

**Figure 2:**
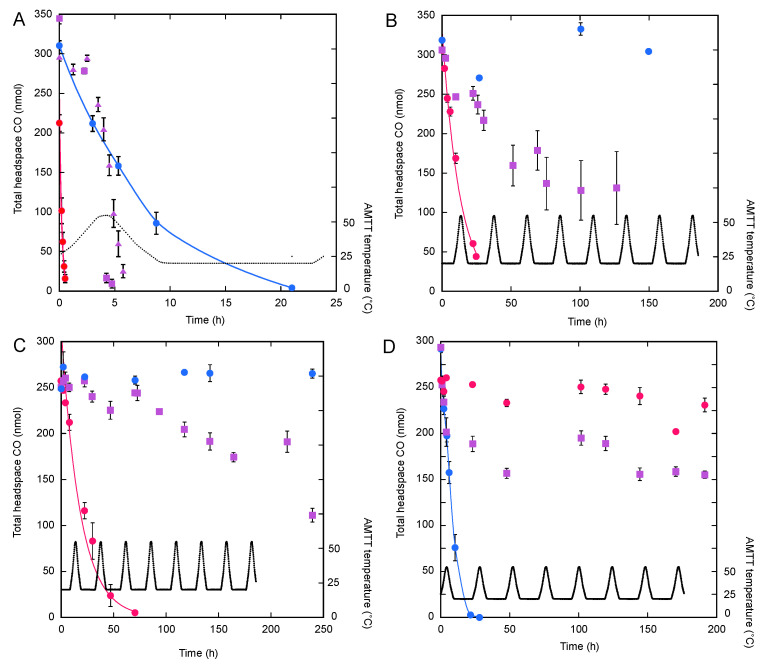
CO uptake by washed cells of *Meiothermus ruber* PS4 (**A**), *Alicyclobacillus macrosporangiidus* CPP55 (**B**), *Thermogemmatispora carboxidovorans* PM5 (**C**) and *Paraburkholderia paradisi* WA (**D**) at 55 °C (•), with the AMTT regime (■) or at 25 °C (•). For *M. ruber* PSR the second CO uptake trial is indicated by (▲). AMTT temperatures are indicated in black. Data are means of triplicate determinations with ±1 standard error; some error bars are smaller than the symbols.

CO uptake assays (Table 2, Figure 2) were conducted using three temperature and two medium treatments (unwashed cells in their growth medium and washed cells in minimal media). *M. ruber* PS4 consumed CO during all incubations (Table 2, Figure 2A). CO uptake rates at 55 °C were 5.5-fold and 16-fold greater than those for the AMTT regime and 25 °C, respectively. Rates for *M. ruber* PS4 substantially exceeded those for all other isolates incubated at 55 *°C* and with the AMTT regime. CO uptake rates by *M. ruber* PS4 were also equivalent to those of mesophilic *P. paradisi* WA for unwashed cell treatments at 25 °C but were exceeded by rates for washed *P. paradisi* WA cells at 25 °C. During the initial AMTT cycle, *M. ruber* PS4 depleted CO rapidly prior to a full 24 h cycle; thus, a second assay was initiated by reamending the same samples the following day. CO uptake rates during the second assay (Figure 2a) were rapid and equivalent to the initial rate.

In contrast to *M. ruber* PS4, the thermophilic spore formers, *A. macrosporangiidus* CPP55 and *T. carboxidovorans* PM5, did not oxidize CO at 25 °C (Table 2). However, similar to *M. ruber* PS4, both isolates were active at 55 °C and in the AMTT treatment (Table 2). CO uptake by the mesophile, *P. paradisi* WA, occurred only during incubations at 25 °C; no activity was observed during the AMTT treatment or at 55 °C (Table 2).

Although CO uptake rates by washed cells were consistently greater than rates for unwashed cells (Table 2), the differences were not statistically significant (*p* > 0.05, unpaired *t*-tests) for any of the three thermophiles during any of the incubations. There was also no difference for washed and unwashed cell CO uptake by *P. paradisi* WA during the AMTT incubation, but rates for washed cells were significantly greater than for unwashed cells at 25 °C (Table 2).

## 4. Discussion

Three phylogenetically and phenotypically distinct thermophilic isolates were able to oxidize exogenous CO (Figure 2) during incubations with multiple cycles of a cyclic temperature regime (AMTT). CO uptake occurred throughout the cycle, including during the mesophilic range, in spite of the limited extent of elevated temperatures (only 3 h d^−1^ ≥ 45 °C). Since *Meiothermus ruber* PS4 also oxidized CO during incubations at 25 °C, activity during the AMTT regime was not surprising. However, no uptake during incubations at 25 °C was observed for *Alicyclobacillus macrosporagiidus* CPP55 or *Thermogemmatispora carboxidovorans* PM5, so the absence of activity under strictly mesophilic conditions does not necessarily predict activity during oscillating mesophilic–thermophilic conditions. Instead, the results suggest that, at least under in vitro conditions, limited periodic exposures to thermophilic temperatures might be sufficient to maintain CO uptake during mesophilic conditions. Under those conditions, CO uptake might promote thermophile survival for those taxa with a capacity to use it (e.g., [30,31]). The absence of a medium effect (similar activity for washed and unwashed cells, Table 2) further suggests that substrate limitation, which often occurs under in situ conditions, might not preclude CO uptake activity. This is consistent with observations for other taxa that CO uptake activity is induced after heterotrophic substrates are depleted (e.g., [32,33]).

In contrast to the three thermophiles, the mesophilic *Paraburkholderia paradisi* WA was unable to sustain CO uptake during a complete AMTT cycle. CO was consumed during the initial mesophilic portion of the cycle, but then activity ceased once temperatures rose into a thermophilic range. Activity did not resume after a return to mesophilic values, indicating that this isolate was likely irreversibly inhibited by a relatively brief exposure to elevated temperatures. However, since mesophiles typically dominate soils that experience brief exposures to elevated temperatures, the behavior of *P. paradisi* WA might not be representative. Of some interest in this context is the fact that *P. paradisi* WA harbors a single 16S rRNA gene [27]. In at least some extreme halophiles and Proteobacteria, multiple rRNA genes have been associated with a capacity for responding to a wide temperature range [34,35]. More detailed analyses of temperature responses by both mesophilic and thermophilic microbes and their adaptations to variable temperature regimes could prove particularly valuable in predicting and understanding microbial community responses to progressive warming, as well as to extreme warming events.

Growth responses were similar to patterns for CO oxidation for the three thermophiles, with the exception that *T. carboxidovorans* PM5 oxidized CO but did not grow during the AMTT regime (e.g., Figure 1 and Figure 2; Table 1 and Table 2). However, the fact that both a spore-forming and non-sporing isolate could grow and oxidize CO during AMTT incubations suggests that at least some thermophilic populations might be maintained in mesophilic soils that experience occasionally permissive temperatures, as proposed by Portillo et al. [20] and Cockell et al. [21]. Thus, microbial transport through the atmosphere and by other mechanisms as well as metabolic activity in situ likely account for patterns of thermophile distribution and persistence in those mesothermal habitats that include occasional elevated temperatures.

The results also suggest that thermophilic communities in mesothermal soils should be comprised of various non-sporing taxa in addition to Firmicutes, although the former might be transport limited. At present, the available data on soil thermophiles are insufficient to test this proposition. Most of the insights available come from cultivation-dependent studies, which have led almost exclusively to reports of spore formers. This might or might not reflect cultivation bias. Cultivation-independent studies of artificially heated mesothermal soils would clearly offer more comprehensive tests (e.g., [16]).

Although the results described here provide support for previous propositions about thermophile activity under mesophilic conditions, it must be noted that a number of important constraints limit extrapolation to soils and in situ activity. Firstly, even if some thermophiles can maintain activity under mesophilic conditions in cultures, activity in situ could be greatly limited by competition with much larger populations of mesophiles that are adapted to ambient conditions. This would include competition for heterotrophic substrates, as well as for CO. Secondly, CO uptake was monitored using concentrations well above atmospheric levels. Therefore, the results are not necessarily indicative of outcomes with ambient CO. Finally, thermophiles might also be constrained during periods of elevated temperatures. Some mesophiles likely remain active between 40–50 °C, and thus, would compete for substrates under those conditions. In addition, elevated temperatures in surface soils might coincide with reduced water potentials that preclude activity by many taxa, including thermophiles (e.g., [36]).

Nonetheless, the collective outcomes from *Meiothermus ruber* PS4, *Alicyclobacillus macrosporagiidus* CPP55 and *Thermogemmatispora carboxidovorans* PM5 during the AMTT cycles suggest that additional emphasis on soil thermophiles is warranted to further explain their distribution and to address their potential contributions to soil carbon cycling in a warmer world. Increases in heat wave frequency, duration and intensity might already have impacted some soil microbial communities [37,38,39]. Future increases in extreme heating could yield temperature regimes that stress mesophiles even more while facilitating activity by some thermophiles.

The biogeochemical roles that thermophilic taxa might play under these conditions are uncertain, but could include contributions to soil–atmosphere CO exchanges. Soil–atmosphere hydrogen exchanges might also be affected. Although little is known about the potential for thermophilic hydrogen uptake by mesothermal soils, the genus *Streptomyces* has been implicated in atmospheric hydrogen consumption [40]. Some members of the genus have been identified as thermophilic hydrogen and CO oxidizers [41]; they and others might function in trace gas dynamics during periods of elevated temperatures when mesophile activity is limited.

## Figures and Tables

**Table 1:** Growth rate constants (k, h^−1^) for isolates incubated at 25 °C, under the AMTT regime or at 55 °C. Values are means of triplicate determinations ±1 standard error.

Isolate	25 °C	AMTT	55 °C
*Meiothermus ruber* PS4	0.05 (0.001)	0.11 (0.01)	0.23 (0.001)
*Alicyclobacillus macrosporangiidus* CPP55	0.0	0.36 (0.001)	0.70 (0.10)
*Thermogemmatispora carboxidovorans* PM5	0.0	0.0	0.41 (0.06)
*Paraburkholderia paradisi* WA	0.33 (0.001)	0.0	0.0

**Table 2:** CO uptake rates (CO nmol h^−1^ (mg protein)^−1^) for each of the three temperature regimes (values are means of triplicate determinations ± 1 standard error) with unwashed and washed cells as described in the text. Asterisks indicate initial uptake rates for *P. paradisi* WA during the AMTT incubations prior to the establishment of thermophilic temperatures.

Strain	Medium	25 °C	AMTT	55 °C
*Meiothermus ruber* PS4	unwashed	2.4 (0.1)	6.5 (0.1)	34.0 (1.0)
	washed	3.1 (0.1)	9.5 (0.2)	53.0 (3.0)
*Alicyclobacillus macrosporangiidus* CPP55	unwashed	0.0	1.5 (0.2)	10.1 (0.5)
	washed	0.0	2.5 (0.6)	15.0 (4.0)
*Thermogemmatispora carboxidovorans* PM5	unwashed	0.0	0.3 (0.01)	3.3 (0.8)
	washed	0.0	0.5 (0.04)	4.3 (0.7)
*Paraburkholderia paradisi* WA	unwashed	2.6 (0.1)	3.0 (1.3) *	0.0
	washed	7.6 (0.5)	3.1 (0.5) *	0.0

## Data Availability

Data are available on request from the authors.
